# The Dangers of Anæsthetics

**Published:** 1897-03-27

**Authors:** 


### The Dangers of Anæsthetics.

The ever-present sense of danger which must exist in the mind of the conscientious anæsthetist while practising his art has lately been stimulated by a suggestion thrown out by Mr. Clement Lucas, who says that in hospital practice the bag and face-piece are often passed from patient to patient without being subjected to sterilisation, and suggests that the lung affections commonly attributed to the anæsthetic may very possibly be due to infected apparatus, instancing a case of pneumonia after operation. We need hardly say that the insinuation so thrown out was immediately and indignantly repudiated by anæsthetists. On the one hand, the fact that pneumonia does follow the administration is broadly denied. Dr. Silk, in a paper read before the Society of Anæsthetists on March 18th, says that, "In the St. Bartholomew's Hospital Reports for 1895 there are tabulated records of 1,774 operations; in those of St. Thomas's Hospital for 1894 there are 1,376; in those of King's College Hospital, Vol. II., 1895-6, there are 915; and in those of Middlesex Hospital, 1894, there are 912. Out of this total of nearly 5,000 I have with some difficulty found references to about thirteen cases of pneumonia; of these no less than eight were jaw or tongue cases, two were in rectal operations, in one a foreign body was subsequently found in the bronchus, and one was a case in which cough and a slight rise in temperature were, apparently, the only signs of pneumonia. Practically, therefore, we have but one case in 5,000 which is at all parallel to the case cited by Mr. Lucas as typical." Others write in the same strain; one gentleman, after stating that every inhaling bag, mask, and face-piece should be thoroughly washed or scalded out, if possible, between each case, adds that with this precaution the addition of the inhaler subjects the patient to no more risk of lung infection "than a ride from Gower Street Station to Portland Road Station on the Metropolitan Railway," and it is maintained generally that if ordinary cleanliness is attended to there is no danger in the use of the mask and the bag. From our observations, however, we are inclined to sympathise with Mr. Lucas; for although we readily admit that many forms of apparatus in common use are capable of being efficiently cleansed, we are not quite so ready to admit that they are as a fact so cleansed between each case, and we think that the attention of instrument makers may be profitably directed to the subject of so constructing the apparatus used in the production of anæsthesia that every portion of it shall be capable not merely of being washed but of being sterilised in a surgical sense. When, however, we turn to another side of the question, and consider, not the suggested dangers attaching to the use of certain forms of apparatus, but the actual dangers of that particular anæsthetic (chloroform) which is so commonly given without any apparatus at all, we find a condition of affairs which is not a matter of doubt or hypothesis but of stern and serious fact. If we depended upon the cases reported lately in the various professional journals, we might imagine that there had been a lull in the monotonous record of deaths from chloroform. This, however, is not the case. We have kept our eyes upon the daily papers, and we find that already in this year, not a quarter of which has yet passed, twenty-two deaths from chloroform have been recorded. It is a ghastly list of catastrophes to happen from the same cause in the course of three months in a country the size of England.

We ask in all seriousness can nothing more be done than is being done to stop this loss of life? In every one of these cases an inquest was held, but the results are extremely unsatisfactory. Verdicts of death from "cardiac failure" in children and young people merely mean that the cause has not been discovered. We have already more than once drawn attention to the fact that in the ordinary methods of chloroform administration commonly in vogue, the so-called open methods, the administrator is necessarily ignorant of the amount of the drug inhaled, and we would ask why a physician who would be so careful about the dose of every other drug should allow himself to be so indifferent about the dose of so potent a drug as chloroform. When we know how small is the dose of chloroform which, if absorbed, will produce anæsthesia—how small even is the dose which will, under the same circumstances, produce death—we cannot but feel astonished that anæsthetists should continue to use the enormous doses which they so often give, or can imagine that they will continue to be held blameless on the plea that only a little of what they give is inhaled by the patient. We fear that so long as the present indifference about dosage continues there will continue to be deaths from chloroform. We will not go so far as to say that the most careful regulation of the dose will in all cases prevent accident, but we do say that an administrator of chloroform would do well to administer it in such a manner as to know how much he is giving.

